# Risk of occult gastrointestinal bleeding with increased gut *Enterococcus* and *Staphylococcus* for poor outcomes in ischemic stroke patients

**DOI:** 10.3389/fnins.2025.1624987

**Published:** 2025-11-17

**Authors:** Geng-Hong Xia, Wei Song, Jia-Hui Xie, Jing-Ru Liang, Jia Yin

**Affiliations:** 1 Department of Neurology, Nanfang Hospital, Southern Medical University, Guangzhou, Guangdong, China; 2 Department of Neurology, Zengcheng Branch of Nanfang Hospital, Southern Medical University, Guangzhou, Guangdong, China

**Keywords:** ischemic stroke, occult gastrointestinal bleeding, gut microbiota, major adverse cardiovascular events, infection-associated pathogens

## Abstract

**Background:**

Gastrointestinal disorders are common in acute ischemic stroke (AIS) patients, but the impact of occult gastrointestinal bleeding (occult GIB) and its link to gut dysbiosis remain underexplored. Occult GIB, often undetected due to subtle symptoms, may significantly affect stroke recovery and long-term outcomes.

**Method:**

We conducted a prospective, multi-center cohort study involving 482 AIS patients. Fecal samples collected within 48 h of admission were analyzed using 16S rRNA gene sequencing. Patients were followed for 1 year to assess major adverse cardiovascular events (MACEs), including death and recurrent stroke.

**Results:**

Occult GIB was identified in 13.9% of patients, who had significantly higher rates of 90-day dependency (56.7% vs. 20.5%) and 1-year MACEs (28.6% vs. 15.5%) compared to non-GIB patients. These patients also exhibited higher infection rates and enrichment of specific gut pathogens, including *Enterococcus*, *Staphylococcus*, and *Pseudomonas* (all *p* < 0.05). Multivariate analysis revealed that elevated levels of these pathogens were independent risk factors for occult GIB. Furthermore, occult GIB independently predicted 90-day dependency (aOR 2.478, 95% CI [1.159–5.296]) and 1-year MACEs (aOR 1.905, 95% CI [1.003–3.617]).

**Conclusion:**

Occult GIB is prevalent in AIS patients and is associated with worse long-term outcomes, particularly in those with enrichment of these specific gut pathogens. Early detection and management of occult GIB may improve patient outcomes. Future research should focus on elucidating underlying mechanisms and developing targeted interventions.

## 1 Introduction

Among non-communicable disorders (NCDs), stroke remains a paramount global health challenge. According to the most recent Global Burden of Disease (GBD) 2021 stroke burden report (Feigin et al., 2024), stroke is the second leading cause of death (accounting for 7.3 million deaths) and the third leading cause of death and disability combined worldwide. The World Stroke Organization’s Global Stroke Fact Sheet 2025 further highlights the escalating burden, noting a 70.0% increase in incident strokes, 44.0% of deaths attributable to stroke, and an 86.0% rise in prevalent strokes. This report underscores that metabolic risks contribute to 69.0% of all strokes, while environmental and behavioral risks constitute 37.0 and 35.0%, respectively (Feigin et al., 2025).

In light of these staggering figures, research has increasingly focused on identifying nontraditional risk factors influencing stroke prognosis, including gastrointestinal (GI) disorders. Notably, GI disorders—particularly those of a functional, inflammatory, or infectious gastrointestinal disorders—have been significantly associated with an elevated risk of ischemic stroke (Roth et al., 2020).

Gastrointestinal bleeding (GIB) is a major complication in patients with acute ischemic stroke (AIS), strongly linked to increased mortality and reduced independence (Roth et al., 2020; Du et al., 2020; Zhou et al., 2019). Evidence demonstrates that GIB is independently elevates the risk of stroke recurrence. A large-scale study of 12,415 ischemic stroke patients revealed that GIB was independently associated with higher recurrence risks at 3, 6, and 12 months post-stroke (Du et al., 2020). GIB is classified into overt GIB (e.g., hematemesis, melena) and occult GIB (detected by fecal occult blood test or iron deficiency anemia). While prior research has predominantly focused on overt GIB and its impact on stroke outcomes and recurrence (Du et al., 2020; Fu, 2019; Aziz et al., 2024), identifying risk factors such as infection, male sex, advanced age, and higher NIHSS scores, proton pump inhibitors (PPIs) have proven effective in reducing the incidence and mortality of overt GIB in AIS patients (Taha et al., 2015).

In contrast, the risks and consequences of occult GIB in stroke outcomes remain poorly understood. Occult GIB is particularly challenging to detect due to its asymptomatic nature and may originate from regions of the GI tract less susceptible to acid suppression (Taha et al., 2015; Raju et al., 2007). The mechanisms linking AIS to occult GIB are likely multifactorial, involving elements such as antiplatelet therapy and stress-induced mucosal injury (Fu, 2019).

Crucially, emerging research implicates gut dysbiosis—a well-documented consequence of ischemic stroke (Yin et al., 2015)—as a potential key player in compromising gastrointestinal integrity. Stroke-induced dysbiosis, characterized by an expansion of pro-inflammatory pathobionts, can increase intestinal permeability, potentially predispose the mucosa to micro-bleeding. Conversely, the presence of intraluminal blood might exacerbate dysbiosis, creating a vicious cycle (Freedberg et al., 2018; Xia et al., 2021; Yin et al., 2015). Ischemic stroke has been shown to rapidly induce pathological alterations in the gut microbiome, as evidenced in both animal models and clinical studies (Yin et al., 2015; Xu et al., 2021). This dysbiosis can, in turn, exacerbate brain infarction through systemic inflammatory pathways, forming a deleterious feedback loop via the brain-gut axis (Xu et al., 2021). Furthermore, gut colonization with specific pathogens, such as *Enterococcus*, has been independently associated with adverse outcomes (including mortality) in critically ill populations (Freedberg et al., 2018; Stein-Thoeringer et al., 2025), and our prior work identified *Enterococcus* as a risk factor for stroke-associated pneumonia (Xia et al., 2021). A recent 2025 study reinforced the role of brain-gut axis dysregulation in post-stroke complications by demonstrating significant alterations in gut microbiota structure and metabolic profiles following acute stroke (Chen et al., 2024).

Although direct evidence establishing a causal or independent correlative link between occult GIB and gut dysbiosis specifically in ischemic stroke patients is still evolving, a compelling body of recent research strongly suggests a plausible and mechanistically supported pathophysiological connection via the brain-gut axis (Xu et al., 2021; Meng et al., 2023; Zhang et al., 2025). While previous studies, including our own, have established stroke-induced gut dysbiosis and its association with systemic complications (Stanley et al., 2016), the specific link between the abundance of gut pathogens and the occurrence of occult GIB during the acute phase of stroke—a common yet underdiagnosed complication—remains markedly understudied. Therefore, to address this significant gap in knowledge, the present study aims to: (1) investigate the association between occult GIB and long-term prognosis in AIS patients, including 90-day dependency and one-year major adverse cardiovascular events (MACEs, including death and recurrent ischemic stroke); (2) explore the association between gut potential pathogens (and their metabolites), and occult GIB; and (3) examine the potential link between increased gut potential pathogens and long-term outcomes.

## 2 Materials and methods

### 2.1 Study population and clinical data collection

**Figure 1:**
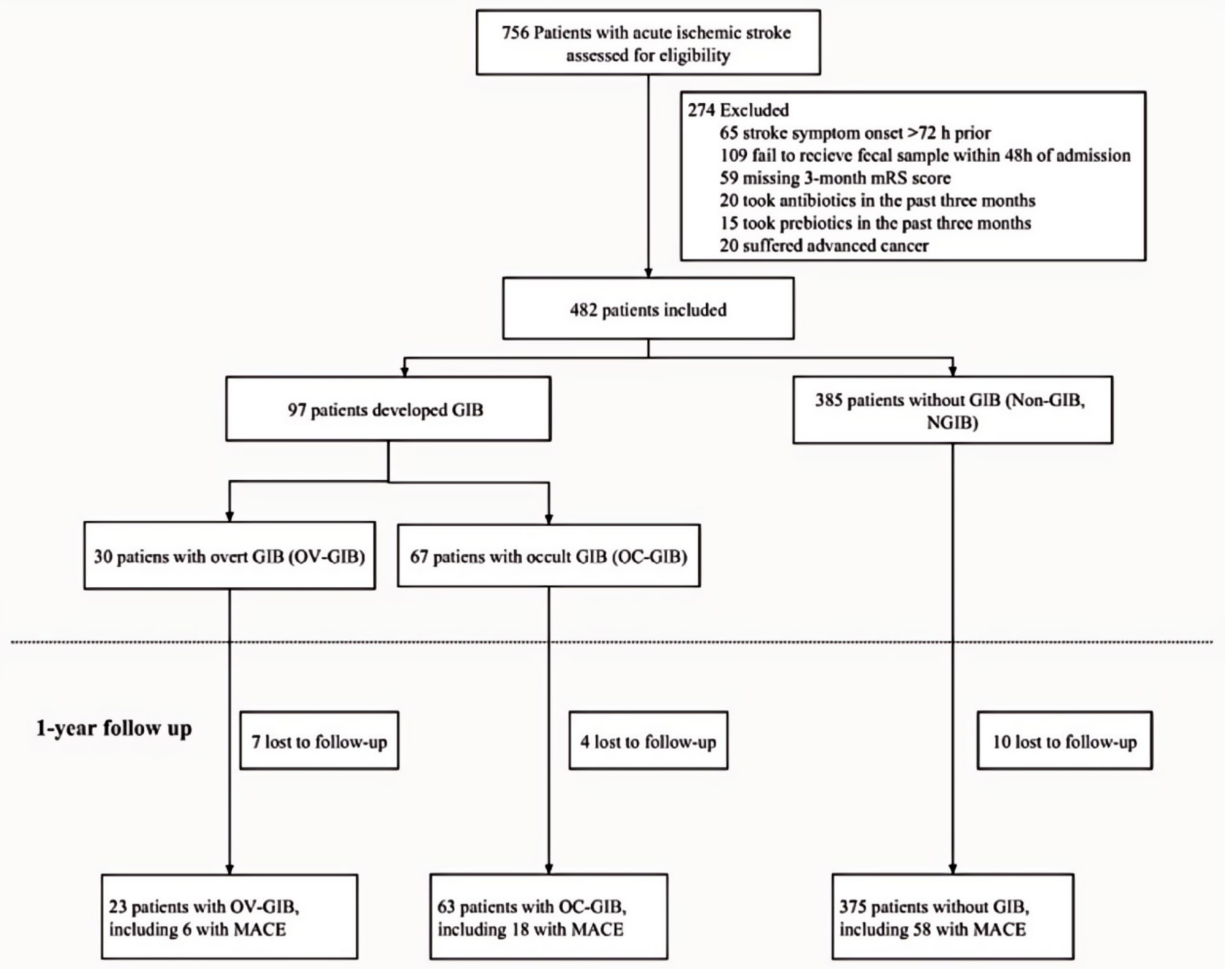
The study flowchart. OC-GIB, occult gastrointestinal bleeding; OV-GIB, overt gastrointestinal bleeding; MACE, major adverse cardiovascular event.

We conducted a prospective, multi-center cohort study involving patients with acute ischemic stroke (AIS) admitted within 72 h of stroke onset to Nanfang Hospital, Yanling Hospital, and Zhujiang Hospital of Southern Medical University from February 2014 to December 2020. Ischemic stroke was defined as a clinical syndrome confirmed by radiographic evidence of an acute infarct on MRI or MRA. The detailed enrollment process is shown in Figure 1.

Inclusion criteria included: (1) age > 18 years; (2) admission within 72 h of stroke onset; (3) ability to provide fresh stool samples within 48 h of admission, (4) availability for three-month follow-up. Exclusion criteria comprised: (1) use of antibiotics, prebiotics, or probiotics within 3 months prior to admission; (2) admission beyond 72 h post-stroke onset; (3) recent gastrointestinal disease symptoms within the past 3 months; (4) presence of gut diseases; (5) active infection within 2 weeks preceding admission; (6) advanced cancer; (7) inability to provide fresh stool samples within 48 h of admission; (8) history of systemic disease such as cirrhosis, renal failure and hematologic disease, or use of an immunosuppressant; (9) lost three-month follow-up. Patients were further followed up to 1 year post-stroke onset.

Demographic and clinical data collected at admission included age, sex, vascular risk factors (hypertension, hyperlipidemia, atrial fibrillation, diabetes mellitus, current smoking, and previous stroke history), initial stroke severity (NIHSS score), dysphagia, use of thrombolytic therapy, and clinical complications (infection and intracranial hemorrhagic event).

Human Ethics and Consent to Participate declarations: The study protocol was approved by the Ethical Committee of Southern Medical University, and informed consent was obtained from all participants or their legal guardians.

The study was conducted in accordance with the Declaration of Helsinki and registered at http://www.chictr.org (ChiCTR-ROC-17011567, registered on 2017-06-05 00:00:00) with the Chinese Clinical Trial Registry.

### 2.2 Definition of overt GIB and occult GIB

Patients underwent fecal occult blood tests (FOBT) within 48 h of admission or gastric juice occult blood test if necessary. Overt and occult GIB events were systematically recorded during the study period. Overt GIB was defined by symptoms such as coffee-ground emesis, hematemesis, blood in the nasogastric tube, or melena that occurred during hospitalization (Du et al., 2020). Occult GIB was identified by a positive fecal occult blood test (FOBT) or iron deficiency anemia with or without a positive FOBT result (Naut, 2016). Diagnoses were confirmed by treating physicians, blinded to other clinical and laboratory data, based on electronic medical records and established criteria.

### 2.3 Clinical outcomes

The primary outcome was defined as an unfavorable functional outcome at 90 days, determined by a modified Rankin Scale (mRS) score of ≥3 within 90 days. The secondary outcomes included: (a) A composite of major adverse cardiovascular events (MACEs) within 1 year post-stroke, including all-cause mortality, myocardial infarction or recurrent ischemic stroke (whichever occurred first). Recurrent ischemic stroke was defined as a new focal neurological deficit of vascular origin lasting >24 h, confirmed by neuroimaging. Outcomes were assessed through structured telephone interviews or clinical record review by assessors blinded to the GIB and microbiota status.

### 2.4 Culture-proven infections

Culture-proven infections within 30 days of admission were recorded and classified by bacteria species, with independent ascertainment blinded to pathogen status by evaluating complete clinical data and using criteria adapted from sepsis-3 (Naut, 2016; Seymour et al., 2016).

### 2.5 Fecal microbiota analysis

Fecal samples collected within 48 h of admission were frozen at −80 °C within 2 hours of collection. DNA extraction and amplification of the bacterial 16S rRNA gene V4 region via polymerase chain reaction (PCR), and subsequent sequencing were performed according to protocols outlined in our previous reports (Xia et al., 2021; Yin et al., 2015; Xia et al., 2019). Microbiota analysis was conducted using QIIME (version 1.9.1), with samples normalized to 8,000 sequences. The association between infection-related pathogens and GIB was estimated by identifying differentially abundant taxa, as described by Freedberg et al. (2018). First, a targeted classification was applied to detect common pathogens, which were classified as present if at least one read was detected; otherwise, they were classified as absent. Operational taxonomic units (OTUs) identifiers were assigned to specific pathogens at the lowest possible hierarchical levels (genus level for *Acinetobacter*, *Klebsiella*, *Staphylococcus*, *Pseudomonas*, and *Enterococcus*). Second, the relative abundance of these specific pathogens within gut microbiota was analyzed.

### 2.6 Serum biomarkers intestinal barrier injury indicators and inflammation-related factors

Fresh serum samples were collected within 24 h of admission. Serum samples were isolated by centrifugation at 3000 rpm for 10 min and stored at −80 °C until testing. Commercial enzyme-linked immunosorbent assay (ELISA) kits (Bioswamp, Myhalic Biotechnology Co., Ltd., Wuhan, China) were used to measure the concentration of intestinal barrier injury indicators in peripheral blood, including lipopolysaccharide (LPS) and lipopolysaccharide-binding protein (LBP), as well as levels of the pro-inflammatory cytokine interleukin (IL)-23 and the anti-inflammatory cytokine IL-10. Standard curves were all within the expected range and all measurements were performed by one experienced staff blinded to the study design.

### 2.7 Statistical analysis

Statistical analyses were performed using SPSS version 24 (IBM SPSS, Chicago, IL). Data are presented as percentages for categorical variables and medians (interquartile ranges) for continuous variables. Continuous variables with skewed distributions were transformed to a log scale. The Mann–Whitney U-test was used for continuous variables, while the *χ*^2^ test or Fisher exact test were used for categorical variables. Subgroup analysis was further conducted, aiming to match participants individually at a 1:1 ratio by age (± 2 years), admission NIHSS (± 2 points), and dysphagia status using propensity score matching.

Potential confounding risk factors for occult GIB, including age, current smoking, dysphagia, initial NIHSS score, white blood cell (WBC) count, hemoglobin levels, and a history of atrial fibrillation (AF) stroke, hypertension, and diabetes mellitus (DM), were analyzed. Variables showing a trend in association with the occult GIB in the univariate analysis (*p* < 0.10) were included in the multivariable model for occult GIB. The variables entered in the multivariate logistic regression model were as follows: male, smoking, dysphagia, a history of atrial fibrillation, hemoglobin levels, initial NIHSS score and glucose. Variables included in multivariate models were chosen based on clinical relevance and association with the outcome in univariate analysis (*p* < 0.10) (90-day outcome model, including occult GIB, NIHSS, age, diabetes, hypertension, AF and *Enterococcus*, 1-year outcome model, including occult GIB, age, hypertension, stroke history and *Staphylococcus*, 1-year recurrent stroke model, including stroke history and *Staphylococcus*). Relative risk was quantified as the odds ratios (ORs) with corresponding 95% confidence interval (CIs). Predictive performance was assessed by comparing receiver operator characteristic (ROC) curves.

## 3 Results

### 3.1 Baseline characteristics of the study population

A total of 482 AIS patients with 3-month follow-up outcomes were included in the final analyses, with 21 patients (4.36%) lost to follow-up by the end of the 1-year observational period. Of these, 97 patients (20.1%) experienced gastrointestinal bleeding (GIB) post-stroke, including 67 (13.9%) with occult GIB and 30 (6.2%) with overt GIB. No significant differences were observed in age, smoking status, diabetes, hypertension, or stroke history among the occult GIB, overt GIB, and non-GIB groups. However, both GIB cohorts had lower hemoglobin levels and higher NIHSS scores compared to the non-GIB group. Admission glucose levels and WBC counts did not significantly differ between the occult GIB and non-GIB groups (Table 1).

**Table 1:** Demographic and clinical characteristics of GIB group and non-GIB group.

Variables	GIB (*n* = 97)		Non-GIB (*n* = 385)	*p* value
	Overt GIB (*n* = 30)	Occult GIB (*n* = 67)		
Demographic features				
Age, Median (IQR), years	67.0 (56.3–74.3)	62.0 (51.0–74.0)	61.0 (50.0–70.0)	0.073
Sex, Male, *n* (%)	22 (73.3)	39 (58.2)	276 (71.7)	0.078
Comorbities and risk factor, *n* (%)				
Diabetes	12 (40.0)	17 (25.4)	111 (28.8)	0.334
Hypertension	24 (80.0)	39 (58.2)	247 (64.2)	0.116
Atrial fibrillation	11 (36.7)^a^	12 (17.9)^b^	25 (6.5)	<0.001
History of stroke	7 (23.3)	15 (22.4)	72 (18.7)	0.687
Current smoking	13 (43.3)	18 (26.9)	152 (39.5)	0.126
Dysphagia	27 (90.0)^a,c^	42 (62.7)^b^	95 (24.7)	<0.001^d^
Baseline clinical status				
Admission serum glucose, Median (IQR), mmol/L	7.4 (5.91–11.00)^a^	6.60 (5.4–8.9)	5.93 (4.82–7.75)	0.002^d^
Admission WBC, Median (IQR), ×10^9^/L	9.9 (8.4–14.3)^a^	8.3 (6.4–10.3)^b^	8.2 (6.6–10.0)	0.002^d^
Admission HGB, Median (IQR), g/L	128.0 (114.5–140.5)^a^	127.0 (119.0–139.0)^b^	139.0 (128.0–151.0)	<0.001^d^
Admission NIHSS score, Median (IQR)	12.5 (7.8–17.3)^a^	11.0 (4.0–17.0)^b^	4.0 (2.0–8.0)	<0.001^d^
Thrombolysis, *n* (%)				<0.001
None	24 (80.0)	45 (67.2)	344 (89.4)	
Intravenous	1 (3.3)	1 (1.5)	16 (4.2)	
Intra-arterial	2 (6.7)	7 (10.4)	11 (2.9)	
Both	3 (1.0)	14 (20.9)	14 (3.6)	

GIB, gastrointestinal bleeding; IQR, interquartile range; NIHSS, National Institutes of Health Stroke Scale; WBC, white blood cell count; HGB, hemoglobin.

a *p* < 0.05 when explained overt GIB group compared with Non-GIB group.

b *p* < 0.05 when explained occult GIB group compared with Non-GIB group.

c *p* < 0.05 when explained overt GIB group compared with occult GIB group.

d *p* < 0.05, significant difference among the three groups.

### 3.2 Poor short-term and long-term clinical outcomes in AIS patients with occult GIB

Compared to non-GIB patients (Table 1), those with occult GIB had significantly higher stroke severity at admission (median NIHSS, [IOR]: 11.0 [4.0–17.0] vs. 4.0 [2.0–8.0], *p* < 0.001). Occult GIB patients were more likely to develop post-stroke complications, including dysphagia (62.7% vs. 24.7%) (Table 1), infection (50.7% vs. 19.5%) and intracranial hemorrhagic events (19.4% vs. 5.2%) (Table 2). Moreover, higher NIHSS (8.0 [2.0–15.3] vs. 2.0 [1.0–5.5], *p* < 0.001) and mRS scores (4.0 [2.0–5.0] vs. 2.0 [1.0–3.0], *p* < 0.001) (Table 2) at discharge were observed in occult GIB patients. The occult GIB group experienced more than twice the rates of 90-day dependency (56.7% vs. 20.5%), 90-day mortality (13.4% vs. 3.1%) as well as higher 1-year MACEs (28.6% vs. 15.5%) compared to the non-GIB group (all *p* < 0.05, Table 2).

**Table 2:** Post-stroke complications and clinical outcomes of GIB group and non-GIB group.

Variables/Outcomes	GIB group (*n* = 97)		Non-GIB (*n* = 385)	*p* value
	Overt GIB (*n* = 30)	Occult GIB (*n* = 67)		
Complication post-stroke, *n* (%)				
Infection	25 (83.3)	34 (50.7)	75 (19.5)	<0.001
Bacteria culture-proven positive	10 (33.3)	21 (31.3)	29 (7.5)	<0.001
Intracranial hemorrhagic event	5 (16.7)	13 (19.4)	20 (5.2)	<0.001
Clinical outcomes				
Short-term outcomes				
NIHSS score at discharge, Median (IQR)	9.0 (4.0–15.5)	8.0 (2.0–15.3)	2.0 (1.0–5.5)	<0.001
mRS score at discharge, Median (IQR)	4.0 (3.0–5.0)	4.0 (2.0–5.0)	2.0 (1.0–3.0)	<0.001
Long-term outcome				
90-day unfavorable outcome (mRS ≥ 3), *n* (%)	26 (86.7)	38 (56.7)	79 (20.5)	<0.001
90-day mortality, *n* (%)	5 (16.7)	9 (13.4)	12 (3.1)	<0.001
One-year mortality, *n* (%)	6/23 (26.1)	12/57 (21.1)	25/342 (7.3)	<0.001
One-year recurrent stroke, *n* /*n*_all_ (%)	0/17 (0)	7/52 (13.5)	35/351 (10.0)	0.328
One-year MACEs, *n* /*n*_all_ (%)	6/23 (26.1)	18/63 (28.6)	58/375 (15.5)	0.022

NIHSS, National Institutes of Health Stroke Scale; mRS, modified Rankin score; MACEs, major adverse cardiovascular events, including death and recurrent stroke. IQR, interquartile range.

Regarding the two subgroups of GIB (occult vs. overt), although the 90-day dependency rate was lower in patients with occult GIB compared to those with overt GIB, similar high rates of 90-day mortality and 1-year MACEs were observed between the two groups (Table 2).

### 3.3 Increased post-stroke infections and enrichment of specific gut pathogens in occult GIB patients

Over half of the occult GIB patients (50.7%) experienced post-stroke infections, 2.5 times higher than non-GIB patients (19.5%) (Table 2). A significantly higher rate of culture-proven pathogens-positive events were noted in occult GIB patients compared to non-GIB patients (31.3% vs. 7.5%, *p* < 0.001) (Table 2). The five most prevalent bacteria species identified were: *Acinetobacter* spp. (32 patients), *Staphylococcus* spp. (16 patients), *Klebsiella* spp. (16 patients), *Pseudomonas* spp. (13 patients), and *Enterococcus* spp. (8 patients).

**Figure 2:**
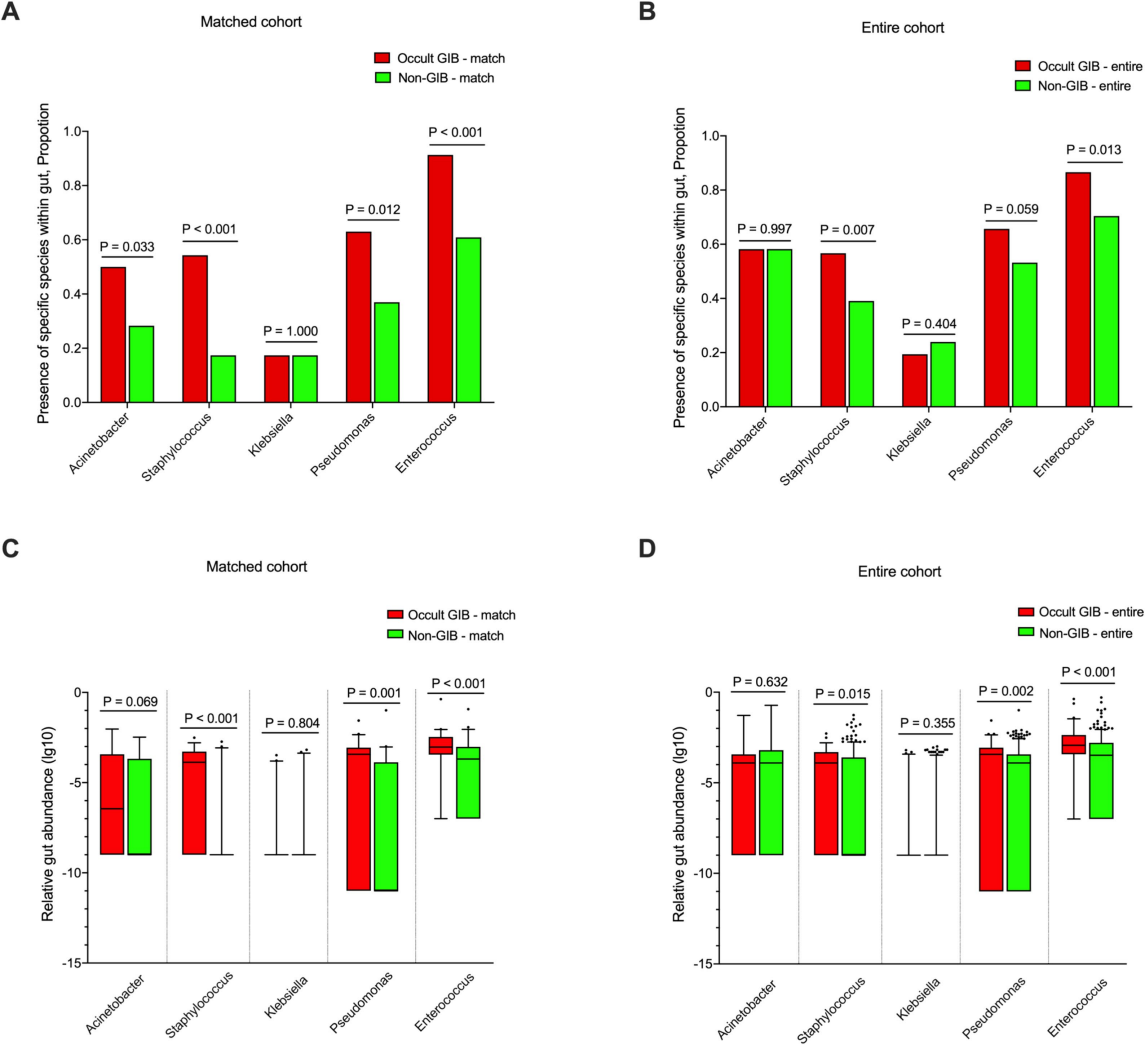
Presence of specific gut pathogens during the acute stage post-stroke in occult GIB and Non-GIB patients, in both the matched cohort and the entire cohort. **(A,B)** The five most common culture-proven infections were from *Acinetobacter* species (spp.), *Staphylococcus* spp., *Klebsiella* spp., *Pseudomonas* spp. and *Enterococcus* spp. Presence of these five most common potential pathogens based on sequencing data in patients with occult GIB compared to patients with Non-GIB in the age-, NIHSS- and dysphagia-matched cohort **(A)** and the entire cohort **(B)**. **(C,D)** Relative abundance of these five potential pathogens in the occult GIB patients and the Non-GIB patients in the matched cohort **(C)** and the entire cohort **(D)**.

To assess the potential association between the abundance of specific gut pathogens and occult GIB, we performed propensity score matching to control for confounding factors such as age, stroke severity (NIHSS), and dysphagia. Patients were individually matched at a 1:1 ratio based on age (± 2 years), admission NIHSS score (± 2 points), and the presence of dysphagia. Ultimately, 46 occult GIB patients and 46 matched non-GIB patients were included (Supplementary Table 1). Fecal samples were collected from these patients within 48 h after admission, and sequencing data were used to compare the presence of five potential pathogens between occult GIB and Non-GIB patients: *Staphylococcus* spp. (54.3% vs. 17.4%, *p* < 0.001), *Pseudomonas* spp. (63.0% vs. 37.0%, *p* = 0.012), *Enterococcus* spp. (91.3% vs. 60.9%, *p* < 0.001), *Acinetobacter* spp. (50.0% vs. 28.3%, *p* = 0.033) and *Klebsiella* spp. (17.4% vs. 17.4%, *p* = 1.000) (Figure 2A). Furthermore, we evaluated the relative abundance of these five genera in the gut. Significantly increased gut abundance of *Staphylococcus*, *Pseudomonas*, and *Enterococcus* (all *p* < 0.05) was observed in occult GIB patients, along with a higher trend for *Acinetobacter* (*p* = 0.069) compared to non-GIB patients in the matched cohort (Figure 2C).

Results were similar in both the whole cohort and the matched cohort, with the presence of *Pseudomonas* spp. (65.7% vs. 53.2%), *Staphylococcus* spp. (56.7% vs. 39.0%) and *Enterococcus* spp. (86.6% vs. 70.4%) being significantly higher in occult GIB patients compared to non-GIB patients (Figure 2B). The gut abundance of *Staphylococcus*, *Pseudomonas*, and *Enterococcus* was also significantly increased in occult GIB patients (all *p* < 0.05) (Figure 2D; Table 3).

**Table 3:** Relative abundance of gut-specific potential pathogens in occult GIB patients compared to non-GIB patients.

Species	Relative abundance		Median (%)	Statistical analysis	
	Occult GIB	Non-GIB	*p* value	Univariate analysis OR (95% CI)	Multivariate analysis aOR* (95% CI)
*Acinetobacter*	0.01	0.01	0.632	ns	ns
*Staphylococcus*	0.01	0.00	0.015	1.122 (1.025–1.228)	1.129 (1.021–1.250)
*Klebsiella*	0.00	0.00	0.355	ns	ns
*Pseudomonas*	0.04	0.01	0.002	1.080 (1.007–1.159)	1.084 (1.002–1.173)
*Enterococcus*	0.12	0.03	<0.001	1.317 (1.114–1.556)	1.256 (1.047–1.507)

*aOR, adjusted with *p* < 0.10 in univariate analysis (male, smoking, AF, dysphagia, hemoglobin, and admission glucose and NIHSS score).

### 3.4 Gut potential pathogens as independent risk factors for occult GIB

Multivariate logistic regression analyses identified gut *Staphylococcus* (aOR, 1.129, 95% CI [1.021–1.250]), *Pseudomonas* (aOR, 1.084, 95% CI [1.002–1.173]), *Enterococcus* (aOR, 1.256, 95% CI [1.047–1.507]) were independently associated with occult GIB, after adjustment for clinical factors, including admission NIHSS and hemoglobin (all *p* < 0.05, Table 3).

**Figure 3:**
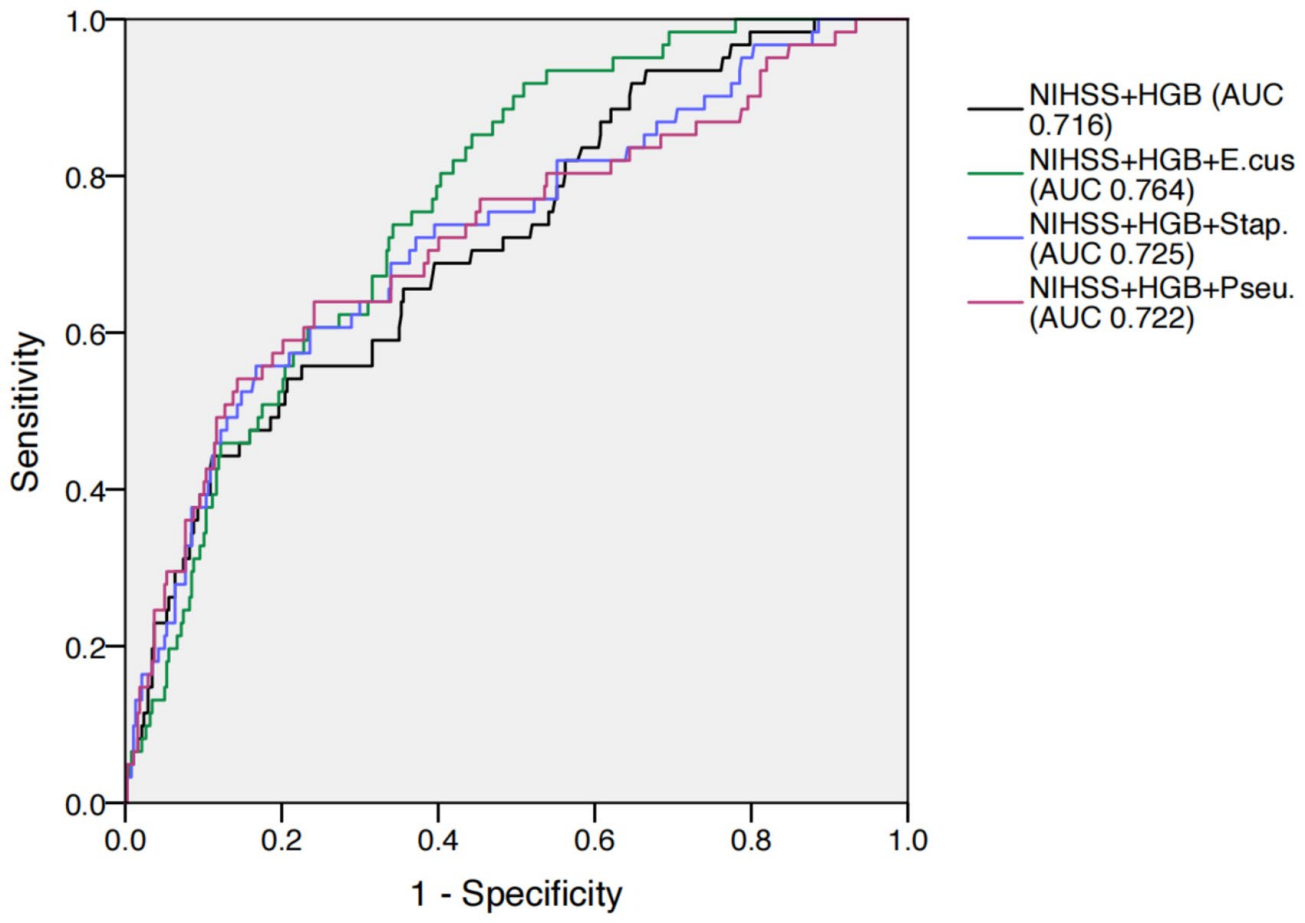
Predictive performance for occult GIB. HGB, homoglobin; E.cus, Enterococcus; Stap., Staphylococcus; Pseu., Pseudomonas. AUC, area under the curve; MDI, microbial dysbiosis index; ROC, receiver operating characteristic.

Receiver operating characteristic (ROC) analysis showed that adding *Enterococcus*, *Staphylococcus*, or *Pseudomonas* separately to the clinical model (admission NIHSS and hemoglobin) significantly improved the area under the curve (AUC) for identifying occult GIB, increasing from 0.716 to 0.764, 0.725, and 0.722, respectively (all *p* < 0.05; Figure 3).

### 3.5 Associations of occult GIB and gut potential pathogens with long-term outcomes in AIS patients

In multivariate logistic regression analyses, occult GIB (aOR 2.478, 95% CI [1.159–5.296]) and gut *Enterococcus* (aOR 1.223, 95% CI [1.052–1.421]) were independent risk factors for 90-day unfavorable outcome (Table 4). Occult GIB was significantly associated with 1-year MACEs (aOR 1.905, 95% CI [1.003–3.617]), but not with recurrent stroke events. Notably, gut *Staphylococcus* was significantly associated with both MACEs (aOR 1.106, 95% CI [1.014–1.206]) and recurrent stroke event (aOR 1.142, 95% CI [1.021–1.278]) (Table 4).

**Table 4:** Univariate and multivariate logistic regression analysis of potential risks for long-term outcomes in AIS patients.

Variables*	Univariate analysis		Multivariate analysis	
	OR (95% CI)	*p* value	aOR (95% CI)	*p* value
90-day unfavorable outcome (mRS ≧3)				
Occult GIB	3.869 (2.274–6.583)	<0.001	2.478 (1.159–5.296)	0.019
NIHSS	1.379 (1.302–1.461)	<0.001	1.370 (1.290–1.454)	<0.001
Age	1.041 (1.025–1.058)	<0.001	1.041 (1.019–1.063)	<0.001
Diabetes	1.630 (1.073–2.477)	0.022	NA	NA
Hypertension	1.496 (0.982–2.279)	0.061	NA	NA
AF	5.290 (2.818–9.929)	<0.001	NA	NA
*Enterococcus*	1.259 (1.122–1.414)	<0.001	1.223 (1.052–1.421)#	0.009
One-year MACEs				
Occult GIB	2.087 (1.136–3.836)	0.018	1.905 (1.003–3.617)	0.049
Age	1.054 (1.032–1.075)	<0.001	1.051 (1.030–1.073)	<0.001
AF	2.694 (1.371–5.297)	0.004	NA	NA
Hypertension	1.820 (1.064–3.115)	0.029	NA	NA
Stroke history	2.080 (1.212–3.569)	0.008	1.846 (1.055–3.231)	0.032
*Staphylococcus*	1.080 (0.994–1.173)	0.070	1.106 (1.014–1.206)†	0.023
One-year recurrent stroke				
Stroke history	2.364 (1.180–4.733)	0.015	2.453 (1.215–4.950)	0.012
*Staphylococcus*	1.136 (1.016–1.269)	0.025	1.142 (1.021–1.278)‡	0.020

Variables included in multivariate models were chosen based on clinical relevance and association with the outcome in univariate analysis (*p* ≤ 0.10). The specific covariates adjusted for in each model are indicated in the table footer (#, †, ‡).

* Variables that demonstrated an association trend in association in the univariate analysis (*p* < 0.10) were included as the covariate factors.

# Adjusted for age, atrial fibrillation, diabetes, hypertension and admission NIHSS score.

† Adjusted for age, atrial fibrillation, hypertension and stroke history.

‡ Adjusted for stroke history.

Occult GIB, occult gastrointestinal bleeding; AF, atrial fibrillation; MACEs, major adverse cardiovascular events. NA, not assessed.

### 3.6 Metabolites and serum biomarkers associated with occult GIB

Patients with GIB exhibited higher serum concentrations of LPS, LBP, and IL-23 and lower concentrations of IL-10 (median [IQR]: LPS, 1608.2 [1081.2–1996.2] pg/mL; LBP, 206.0 [148.9–252.2] ng/mL; IL-23, 567.7 [447.6–716.7] pg/mL; IL-10, 87.5 [65.2–110.6] pg/mL), similar to those observed in patients with occult GIB (LPS, 1576.5 [1164.6–2049.4] pg/mL; LBP, 196.2 [149.2–237.1] ng/mL; IL-23, 568.0 [437.3–722.5] pg/mL; IL-10, 90.2 [64.6–112.5] pg/mL), compared to patients without GIB (LPS,1297.4 [1043.8–1611.0] pg/mL; LBP, 171.9 [125.2–221.4] ng/mL; IL-23, 484.7 [383.1–646.9] pg/mL; IL-10, 96.4 [77.9–120.1] pg/mL) (Supplementary Figure 1). These biomarker profile, obtained from a single early measurement, is consistent with a state of systemic inflammation and potentially reflects compromised intestinal mucosal integrity in patients with occult GIB compared to those without GIB.

## 4 Discussion

This study diverges from previous research predominantly focusing on overt gastrointestinal bleeding (GIB) post-stroke, by examining the impacts of occult GIB on long-term outcomes in acute ischemic stroke (AIS) patients and revealing its association with an enrichment of specific gut pathogens. Our findings revealed that occult GIB is highly prevalent in 13.9% of AIS patients, significantly higher than overt GIB (6.2%). Compared to non-GIB patients, those with occult GIB exhibited higher rates of bacterial infections and a heightened abundance of specific gut pathogens, including *Enterococcus*, *Staphylococcus*, and *Pseudomonas*, which were identified as independent risk factors for occult GIB. Elevated gut permeability biomarkers, (LBP and LPS) and a more severe inflammatory profile (higher IL-23, lower IL-10) were observed in occult GIB patients, correlating with worse outcomes, including higher 90-day dependency and 1-year major adverse cardiovascular events (MACEs).

Gastrointestinal bleeding is a significant contributor to increased mortality in stroke patients, with much attention historically focused on the treatment and prevention of overt GIB. In our study, the prevalence of overt GIB was 6.2%, aligning with prior studies where GIB incidence ranged from 1.24 to 8.1% (Du et al., 2020). Notably, our multi-center cohort study highlighted a rising incidence of occult GIB in AIS patients, revealing distinct clinical characteristics and long-term outcomes compare to both overt GIB patients and non-GIB patients. Lower admission hemoglobin levels were observed in both GIB groups, consistent with previous findings (Taha et al., 2015). Patients with overt GIB exhibited higher admission WBC levels and were more likely to experience dysphagia, infections, and 90-day dependency compared to those with occult GIB or without GIB. However, at the one-year follow-up, patients with occult GIB had similar mortality and MACEs rates to those with overt GIB. Occult GIB post-stroke was identified as an independent risk factor for 90-day unfavorable outcomes and one-year MACEs after adjusting for traditional vascular risk factors in AIS patients. These findings highlights the need for vigilant management of occult GIB post-stroke and long-term care of these patients. While this study cannot establish a cause-effect relationship, these results have important clinical implications.

The underlying mechanism linking AIS and occult GIB remains unclear. Proposed mechanisms contributing to mucosal injury after ischemic stroke include antiplatelet use, stress, vagal hyperactivity (Fu, 2019) and disruption of the brain-gut axis, which may increase gastrointestinal mucosal injury risk. Some preventive strategy, including selective NSAIDs such as coxibs (Taha et al., 2015), or other antithrombotic drugs, appear less likely to cause mucosal damage in the lower gastrointestinal tract. However, managing occult GIB solely with acid inhibitors may be unrealistic, given the multiple etiological factors and the complex anatomical sites. These drugs are only effective for lesions in acid-susceptible areas such as the esophagus, stomach, or duodenum. Interestingly, researchers have found that the incidence of occult GIB was significantly correlated with the prescriptions of proton pump inhibitors (PPI) (Taha et al., 2015). Post-stroke alternations in the gut microbiome, particularly the enrichment of pathogenic species, may also contribute to occult GIB development. Recent research, including our previous studies (Yin et al., 2015; Xu et al., 2021; Xia et al., 2019), have demonstrated that ischemic stroke induces pathological alternations in the host gut microbiome, as evidenced in both animal and clinical studies. Increased pathogenic species and associated metabolites have been reported to increase the risk of ischemic stroke [e.g., atherosclerosis, obesity, diabetes mellitus, and hypertension (Boulangé et al., 2016; John and Mullin, 2016; Mazidi et al., 2016)] and post-stroke complications [e.g., pneumonia (Stanley et al., 2016; Stevens et al., 2022; Fu et al., 2024), and cognitive impairment (Olson et al., 2021; Pan et al., 2023; Wang et al., 2023)]. In our previous study, patients with stroke-associated pneumonia (SAP) exhibited increased gut microbiota dysbiosis in the acute stage, with *Enterococcus* identified as an independent risk factor of SAP (Xia et al., 2021). Increased abundance of gut pathogenic species, including *Enterococcus*, *Pseudomonas*, *Staphylococcus*, and *Klebsiella*, were positively associated with specific bacteria infection and even mortality, as confirmed by stool or swab culturesand 16S sequencing (Freedberg et al., 2018; Shimasaki et al., 2019). In this study, we observed that patients with occult GIB had a significantly higher risk of infection, with culture-proven bacteria events. We further found that certain pathogens, including *Enterococcus*, *Pseudomonas*, and *Staphylococcus*, were more prevalent in occult GIB patients during the early acute stage, both in the age-, dysphagia- and NIHSS- matched cohort, as well as in the entire AIS patient cohorts. Multivariate logistic regression analysis confirmed that these pathogens were independent risk factors for occult GIB in AIS patients. Our study identified specific pathogens (*Enterococcus*, *Staphylococcus*, *Pseudomonas*) that were independently associated with occult GIB. This association raises the possibility of a link between this specific pathogen enrichment and gastrointestinal mucosal integrity post-stroke, though the direction of this relationship requires further investigation. Furthermore, the co-occurrence of gut pathobionts and systemic infections suggests a shared underlying dysbiotic state or potential cross-talk, but our study design cannot confirm a direct gut origin for post-stroke infections. We also assessed the biomarkers of gut permeability and inflammation in patients. The systemic biomarker profile observed in the early acute phase—elevated LBP, LPS, and IL-23, and reduced IL-10—in occult GIB patients is indicative of a heightened systemic inflammatory state. While this pattern could be consistent with increased gut permeability and mucosal injury, it is crucial to note that these single early measurements may also reflect, and are likely confounded by, the acute systemic inflammatory response to the stroke itself. In terms of long-term outcomes, increased *Enterococcus* were independently associated with a higher risk of 90-day dependency while increased *Staphylococcus* was linked to one-year MACEs and recurrent stroke. However, the direct association between these biomarkers and occult GIB was not fully elucidated in this study. Targeting these enriched gut pathobionts and gut-derived metabolites may help to improve GIB management and enhance long-term outcomes.

Our study has limitations. First, we did not monitor longitudinal changes in gut pathogens and occult GIB over time, or explore the underling mechanisms linking gut pathogens to adverse outcomes, limiting our ability to established causality. Secondly, our analysis was focused on a pre-selected set of pathogens based on their clinical relevance. We did not perform *α*- and *β*-diversity analyses to characterize the overall gut microbial community structure. Therefore, our findings should be interpreted as demonstrating an enrichment of specific pathogens associated with occult GIB, rather than providing a complete picture of global gut dysbiosis. Future studies including comprehensive community-level analyses are warranted. Additionally, our sample size may limit the statistical power to detect certain associations. Furthermore, due to the cross-sectional nature of the microbiota assessment at admission, we cannot establish causality or rule out reverse causality—whether occult GIB (or its predisposing conditions) alters the gut environment promoting pathogen growth, or whether pre-existing or stroke-induced dysbiosis contributes to mucosal vulnerability leading to occult GIB. Further research should focus on elucidating these mechanisms and developing targeting interventions to manage gut pathogens and improve outcomes in AIS patients.

## 5 Conclusion

In this prospective multicenter study, occult GIB was prevalent in nearly 14% of AIS patients and was associated with significantly worse short- and long-term functional outcomes and survival. The enrichment of specific gut pathogens (*Enterococcus*, *Staphylococcus*, *Pseudomonas*) in the acute phase was independently associated with occult GIB. These findings highlight occult GIB as a clinically significant yet often overlooked complication of AIS. The association with this specific gut pathogen profile warrants further investigation to elucidate potential causal mechanisms and explore targeted interventions aimed at modulating the gut microbiome to improve stroke outcomes.

## Data Availability

The relative-abundance matrix of gut pathogens and corresponding clinical metadata are publicly available via Zenodo: Xia (2025). Gut pathogens in 482 stroke patients [Data set]. Zenodo. https://doi.org/10.5281/zenodo.17470370.
