# Supplementary material for: Risk of occult gastrointestinal bleeding with increased gut Enterococcus and Staphylococcus for poor outcomes in ischemic stroke patients
Source: Front Neurosci. 2025 Nov 17;19:1624987. doi: 10.3389/fnins.2025.1624987 (PMC12665750; doi:10.3389/fnins.2025.1624987)
Supplement: Supplementary file 1 [file Data_Sheet_1.pdf]

Supplemental Table 1

Occult GIB patients and NIHSS-, age and dysphagia- matched patients without GIB.

	Occult GIB-match group (n=46)	Non-GIB-match group (n=46)	P value
Age, Mean (SD), years	62.0 (13.62)	62.3 (13.56)	0.909
Dysphagia, n(%)	22	22	1.000
admission NIHSS score, Median (IQR)	5.0 (2.00 - 13.00)	5.0 (2.75 - 12.00)	0.875
Presence of gut species, n(%)			
Acinetobacter	23 (50.0)	13 (28.3)	0.033
Staphylococcus	25 (54.3)	8 (17.4)	<0.001
Klebsiella	8 (17.4)	8 (17.4)	1.000
Pseudomonas	29 (63.0)	17 (37.0)	0.012
Enterococcus	42 (91.3)	28 (60.9)	<0.001

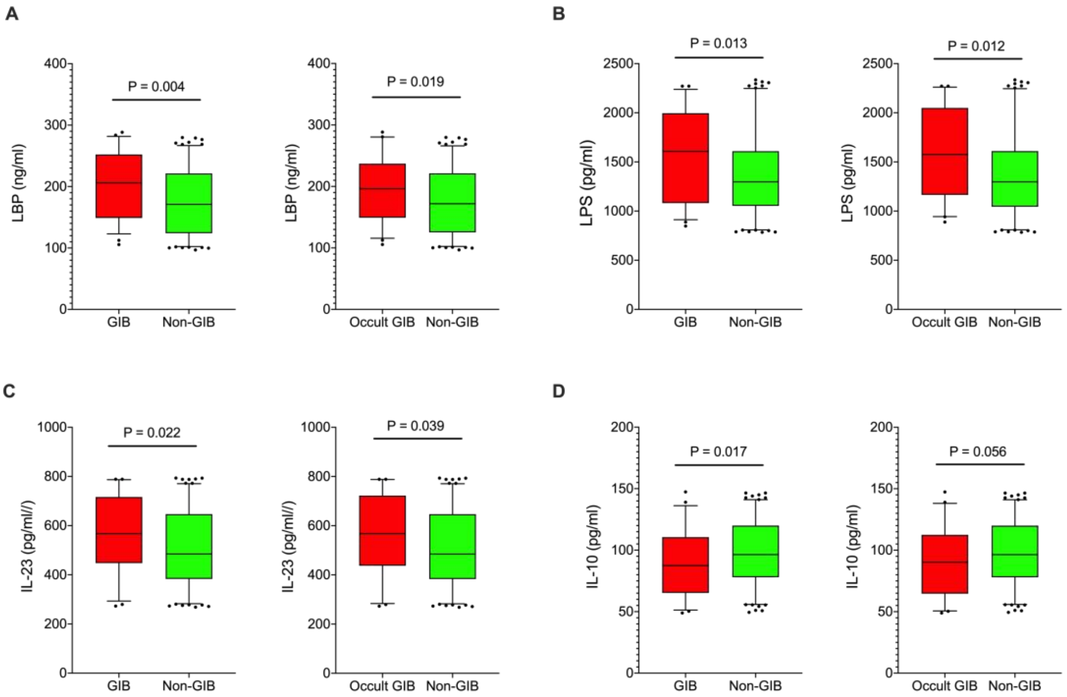

Fig. S1 Comparison of serum intestinal integrity and inflammatory biomarkers between GIB and Non-GIB patients. Patients with GIB (A-B, left) or patients with occult GIB (A-B, right) had increased serum LBP (A) and LPS levels (B) at the early acute stage than patients without GIB (A-B). Elevated serum IL-23 (C) and lower serum IL-10 (D) were observed in patients with GIB, as well as in occult GIB, with comparison to patients without GIB (Non-GIB).
